# Eslicarbazepine Acetate Modulates EEG Activity and Connectivity in Focal Epilepsy

**DOI:** 10.3389/fneur.2018.01054

**Published:** 2018-12-11

**Authors:** Giovanni Pellegrino, Oriano Mecarelli, Patrizia Pulitano, Mario Tombini, Lorenzo Ricci, Jacopo Lanzone, Marianna Brienza, Chiara Davassi, Vincenzo Di Lazzaro, Giovanni Assenza

**Affiliations:** ^1^San Camillo Hospital IRCCS, Venice, Italy; ^2^Department of Human Neurosciences, Sapienza University, Policlinico Umberto I Hospital, Rome, Italy; ^3^Neurology Department, Campus Biomedico University of Rome, Rome, Italy

**Keywords:** eslicarbazepine, EEG, antiepileptic drugs, epilepsy, connectivity, focal epilepsy, networks, power

## Abstract

**Introduction:** Eslicarbazepine acetate (ESL) is an antiepileptic drug approved as monotherapy or add-on for the treatment of epilepsy with seizures of focal onset. ESL owns a good profile in terms of efficacy and tolerability, but its effects on EEG activity and connectivity are unknown. The purpose of this study was to investigate EEG activity and connectivity changes after ESL treatment in persons with focal epilepsy (PFE).

**Material and Methods:** We performed a multicentre, longitudinal, retrospective, quantitative EEG study on a population of 22 PFE, and a group of 40 controls. We investigated the ESL-related changes of EEG power spectral activity and global connectivity [phase locking value (PLV), amplitude envelope correlation (AEC) and amplitude envelope correlation of orthogonalized signals (Ortho-AEC)] for standard frequency bands (delta to gamma). Seizure frequency was evaluated to assess ESL efficacy in our cohort.

**Results:** ESL significantly enhanced both global power spectral density and connectivity for all frequency bands, similarly for all connectivity measures. When compared to the control group, Post-ESL power was significantly higher in theta and gamma band. Pre-ESL connectivity values were significantly lower than control for all frequency bands. Post-ESL connectivity increased and the gap between the two groups was no longer significant. ESL induced a 52.7 ± 41.1% reduction of seizure frequency, with 55% of clinical responders (reduction of seizures ≥50%).

**Discussion:** ESL therapy induces significant enhancement of brain activity and connectivity. Post-ESL connectivity profile of epilepsy patients was similar to the one of healthy controls.

## Introduction

Eslicarbazepine (ESL) is an antiepileptic drug (AED) approved for the treatment of epilepsy with seizures of focal origin (1). ESL is more effective than placebo as add-on therapy (2), is effective and safe in monotherapy in patients with uncontrolled partial-onset seizures (3–5) and has better side effects profile when compared to carbamazepine and oxcarbazepine (6).

The molecular mechanism on which ESL relies to express its therapeutic action is mainly related to the inactivation of voltage-gated sodium channels, similarly to oxcarbazepine, and carbamazepine (7). This mechanism is very specific, as ESL does not bind the receptors for benzodiazepine, GABA and glutamate (8). Differences between ESL and oxcarbazepine and carbamazepine depend on a variety of factors, but largely on the pharmacokinetic profile (7).

Very little is known on the effects of this medication on the brain cortex as a system. AEDs have an effect on brain networks and although the literature is poor of studies explicitly addressing this topic, there is plenty of evidence that antiepileptic medications influence cortical rhythms and networks as measured by EEG (9–14).

EEG is a sensitive and reliable tool to substantiate cortical function. In particular, each frequency band power (alpha, beta, theta, delta, gamma) owns a functional significance. In epilepsy patients, the study of rapid rhythms can help in better understanding positive AED effects of cognition, attention, perception and language networks. On the opposite, the study of slow frequency bands allows to detect effects on memory, drowsiness and cortical plasticity [for a review see Assenza et al. (15)]. In a recent study our group demonstrated that ESL is an AED with a very good efficacy against seizures with a positive impact on sleepiness, depressive symptoms and quality of life (16).

Here we have performed a multicentric, longitudinal study to investigate the impact of ESL on cortical activity and connectivity, as explored by quantitative EEG, in a population of persons with seizures with focal onset (PFE).

## Materials and Methods

### Patients and Study Design

Twenty-two PFE (ILAE definition, mean age = 46 ± 16.7 (SD); 46% females) and a control group of forty healthy subjects (mean age = 50.6 ± 19.04; 45% female, *p* > 0.200 consistently) were enrolled at the epilepsy clinic of Department of Human Neurosciences enrolled of Policlinco Umberto I Universitary Hospital of Rome and of Campus Bio-Medico University of Rome. The study was approved by the ethic committee of Policlinco Umberto I Ethic Board—Rome—and Campus Biomedico University Ethic Board—Rome-. All patients signed a written informed consent. All procedures were performed in agreement with the 1964 Helsinki declaration and its later amendments.

For the epilepsy group the inclusion and exclusion criteria were the following:

Inclusion:

- PFE > 18 years-old;
- two clinical EEG recordings occurring immediately before (< 30 days) ESL assumption and after at least 3 months of ESL assumption;
- resting state EEG free of relevant artifact > 5 min.

Exclusion:

- PFE taking neuroactive drugs other than antiepileptic drugs;
- antiepileptic medication titration in between the two EEG recordings.
- Clinical seizures in the 24 h before EEGs

Healthy subjects were enrolled among the relatives of patients investigated at the outpatient clinic, hospital personnel, and volunteers and were interviewed by a neurologist to rule out medical conditions potentially biasing the study. Patients underwent standard clinical EEG recording before starting eslicarbazepine acetate and after a 3 months period of follow-up. Detailed clinical info is reported in Table S1.

### EEG Recording and Analysis

Nineteen channel-EEG was acquired with a Micromed recorder (Micromed, Mogliano Veneto, IT) between 11:00 and 13:00 a.m. and between 16:00 and 18:00 p.m to minimize drowsiness. The electrodes were placed according to the international 10–20 system (Fp1, Fp2, F3, F4, C3, C4, P3, P4, F7, F8, T3, T4, T5, T6, O1, O2, Fz, Cz, Pz). The reference was placed on FPz and the ground on FCz. Impedance was kept below 5 kOhm for all electrodes. The sampling rate was set to 256 Hz. Eye blinks, eye movements and electrocardiogram (EKG) were recorded using dedicated bipolar electrodes. The resting EEG recording lasted 5 min and was performed with patients with closed eyes, seated on a comfortable armchair in a quiet room. Patients were instructed as follows: “Free your mind, do not think about anything and just relax.” Subjects were also asked to keep their regular wake/sleep cycle before participation. Pre ad Post medication EEGs were measured always using the same apparatus.

EEG signals were analyzed using the EEGLab Toolbox (17), the Brainstorm Toolbox (18) for Matlab (The Math Works Inc, Natick, MA), and in home Matlab code. Both EEGLab and Brainstorm are documented and freely available for download online under the GNU general public license (https://sccn.ucsd.edu/eeglab/ and http://neuroimage.usc.edu/brainstorm). EEG pre-processing consisted of: (a) visual inspection for rejection of possible electrographic seizures, interictal epileptic activity, bad channels, (b) [1–70Hz] band-pass filter, (c) [50Hz] notch filter, (d) Independent Component Analysis (ICA) (19) to remove artifacts related to heartbeat and eye movements, (e) spline interpolation of previously rejected bad channels, (f) re-reference to average reference (20, 21).

Epochs containing interictal epileptic activity (spikes, spike and wave complexes, spiky waves, bouffè of monomorphic slow waves) were rejected. Those PFE with one EEG recording containing more than 20% of interictal epileptic activity were excluded (22).

#### Brain Network Analysis: Resting State Activity and Connectivity

To assess the effect of ESL on brain networks we performed measures of resting state brain activity and connectivity. As measure of activity, we computed the Power Spectrum Density (PSD) by standard FFT approach (Welch procedure: average of non-overlapped windows with a duration of 2 s) for the following frequency bands (delta: 2–4 Hz; theta: 5–7 Hz, alpha: 8–12, Hz, beta1: 13–20 Hz, beta2: 21–29 Hz, gamma: 30–60 Hz). To obtain a measure of global activity, we averaged PSD measures over all channels.

As for connectivity, we focused on the Phase Locking Value (PLV). Phase Locking Value is a very popular measure of non-directional frequency-specific synchronization reflecting long-range integrations. It assesses the extent to which the phase difference between two signals changes over time and is a measure of cortical synchronization based on phase covariance between signals. PLV owns a good time resolution and provides insight on the synchronization of fast changing electrophysiological dynamic. High PLV indicates higher synchronization (23–27). We measured PLV for all possible channel combinations and averaged to obtain a measure of global connectivity. We focused on the same frequency bands as for brain activity (namely, delta: 2–4 Hz; theta: 5–7 Hz, alpha: 8–12, Hz, beta1: 13–20 Hz, beta2: 21–29 Hz, gamma: 30–60 Hz).

The low number of sensors and the detrimental effect of volume conduction on estimation of EEG connectivity might be an issue when assessing connectivity between sensors or brain regions (28). Therefore, we also estimated as additional measures of cortical connectivity. In particular we focused on the Amplitude Envelope Correlation (AEC) and its variant estimated on orthogonalized signals (Ortho-AEC) that regresses out lag = 0 synchrony and minimizes volume conduction issues. To be noted, all connectivity measures estimated after orthogonalization do not only remove the influence of volume conduction, but also the true lag = 0 connectivity. Both AEC and Ortho-AEC capture different features of the connectivity pattern as compared to PLV (29). In further details, AEC measures the linear correlations between the amplitude of the envelops of band-pass filtered signals, relies on the temporal dynamics of relative slow power fluctuations and provides an EEG connectivity measure resembling—at least on some extent—connectivity patterns unveiled by fMRI analysis of BOLD signal (30–33). Finally, to further get rid of the volume conduction bias, we focused on global connectivity and on a within subject pre-post medication paired comparison.

### Clinical and Behavioral Assessment

ESL efficacy was measured on the percentage of seizures reduction. We defined as responders those patients with seizure reduction ≥50% and non-responders the remaining ones.

As EEG activity is very sensitive to sleepiness and cognitive performance (19), we searched in patients' files for the Epworth Sleepiness Scale (ESS) and Stanford sleepiness scale to assess sleepiness and the Beck Depression Inventory-II (BDI) scale to assess depression (34, 35). Sleepiness and depression scores were available for 12 out of 22 patients.

### Statistical Analysis

Statistical analysis was performed using the IBM SPSS Statistics (Ver. 24) and Matlab (Mathworks). Data distribution was checked by means of Kolmogorov-Smirnov test. The effect of ESL on global cortical power and connectivity within the epilepsy group was tested with a repeated measure ANOVA with *Band* (six levels: Delta, Theta, Alpha, Beta1, Beta2, Gamma) and Time (two levels: Pre-ESL and Post-ESL) as within subject factors. In order to compare the epilepsy group and the control group, we applied a mixed model repeated measure ANOVA, with *Band* (six levels: Delta, Theta, Alpha, Beta1, Beta2, Gamma) as within subject factor and *Group* two levels: Control and Pre*-*ESL (or Post*-*ESL) as between subjects factor. The Greenhouse-Geisser correction was applied when needed. A paired *t*-test was used to study changes of behavioral scale scores. The significance level was set to alpha = 0.05.

## Results

### Pre-post Comparison Within the Epilepsy Group

#### EEG Activity

**Figure 1:**
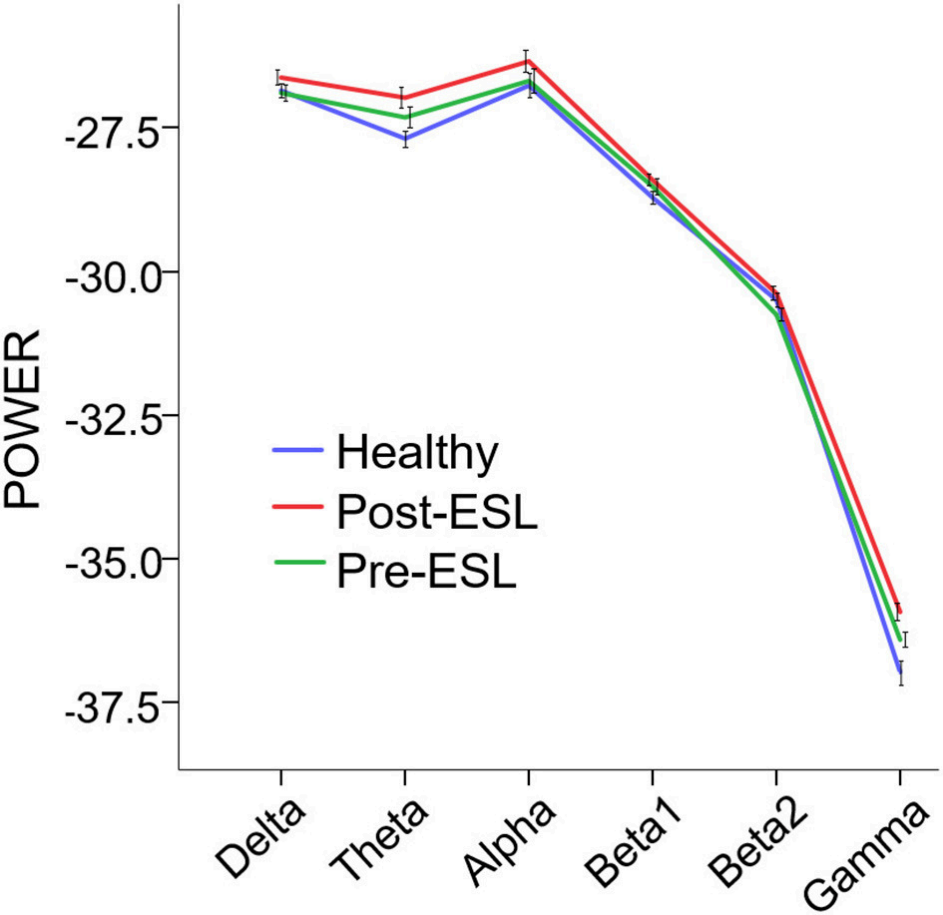
Power Spectral Density (PSD) Pre and Post eslicarbazepine acetate and for the control group. PSD is expressed as average across channels. Error bars represent standard error of the mean. PSD was globally higher post-eslicarbazepine acetate than before.

The repeated measure ANOVA showed a significant main effect of the factor *Time* [*F*_(1, 21)_ = 7.288, *p* = 0.013], suggesting a global increase of connectivity after ESL. No significant *Band by Time* interaction was found [*F*_(3.081, 64.692)_ = 2.657, *p* = 0.054], ruling out a specific ESL effect for selected frequency bands (Figure 1). A significant main effect of the factor *Band* [*F*_(2.598, 54.551)_ = 1077.993, *p* < 0.001] was also found, suggesting an expected difference in power across bands.

#### EEG Connectivity

**Figure 2:**
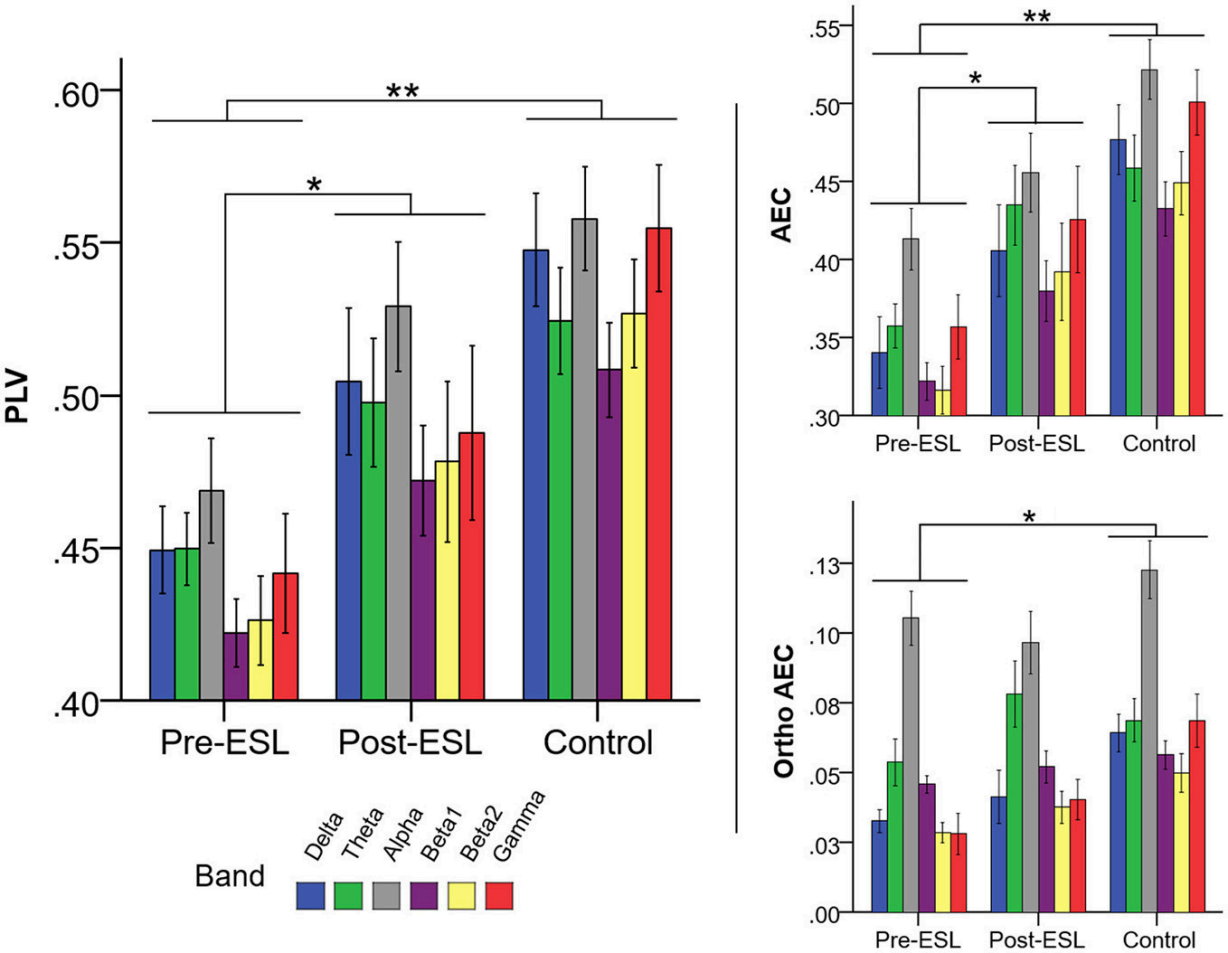
Connectivity Pre and Post eslicarbazepine acetate and for the control group. Left panel shows PLV, right panel shows AEC, and AEC computed on orthogonalized data. Connectivity was higher post-eslicarbazepine acetate than before. Pre-ESL connectivity values were significantly lower than control for all frequency bands. Post-ESL connectivity increased and the gap between the two groups was no longer significant. A similar pattern was observed for all connectivity measures under investigation. *denotes *p* < 0.05; **denotes *p* < 0.001.

The repeated measure ANOVA revealed a significant main effect of the factor *Time* [*F*_(1, 21)_ = 4.359, *p* = 0.049] related to a global increase of connectivity after ESL (Figure 2). No significant *Band by Time* interaction was found [*F*_(2.702, 56.736)_ = 0.196, *p* = 0.881], ruling out a specific ESL effect for selected frequency bands. A significant *Band* main effect [*F*_(2.088, 43.843)_ = 6.101, *p* = 0.004] confirmed the expected connectivity modulation over frequency bands. Similar results were found on different measures of brain connectivity. For AEC a significant factor *Time* [*F*_(1, 21)_ = 5.277, *p* = 0.032], together with lack of *Band* by *Time* interaction, suggested a global higher connectivity level *Post-ESL* as compared to *Pre-ESL*. A similar trend, although without reaching statistical significance, was revealed by the analysis of the orthogonalized version of AEC [Factor Time: *F*_(1, 21)_ = 2.527, *p* = 0.127] (Figure 2).

### Comparison Between Epilepsy and Control Groups

#### EEG Activity

The comparison between *Pre-ESL* and controls revealed no significant difference between groups [Factor *Group*: *F*_(1, 60)_ = 0.695, *p* > 0.200], but a significant *Band* by *Group* interaction [*F*_(5, 300)_ = 3.298, *p* = 0.006]. The *post-hoc* tests, however, did not reveal any significant difference after Bonferroni correction. The comparison between *Post-ESL* and *controls* also revealed a significant *Band* by *Group* interaction [*F*_(5, 300)_ = 4.979, *p* < 0.001] related to a significantly higher *Post-ESL* power for *Theta* and *Gamma* bands (Bonferroni-corrected *post-hoc* comparisons *p* = 0.018 and *p* = 0.06 respectively).

#### EEG Connectivity

The Comparison of PLV values between *Pre-ESL* and Controls revealed a significant factor *Group* [*F*_(1, 60)_ = 15.565, *p* < 001] and no interaction, suggesting that PLV connectivity was significantly higher in the controls as compared to epilepsy patients before ESL administration. Notably, no significant *Group* difference nor interactions were found when comparing Controls vs. *Post-ESL* [main factor *p* = 0.129, interaction factor *p* > 0.200], suggesting that ESL reduced the gap between the epilepsy group and controls for all frequency bands (Figure 2).

The same profile was confirmed for the other two measures of brain connectivity, namely AEC, and its orthogonalized version. Indeed, in all cases the comparison between Controls and Pre-ESL revealed a significant *Group* factor and no significant interactions [AEC: Factor *Group F*_(1, 60)_ = 22.815, *p* < 0.001, *Group* by *Band* interaction *p* > 0.200; Orthogonalized AEC: Factor *Group F*_(1, 60)_ = 10.281, *p* = 0.002, *Group* by *Band* interaction *p* > 0.200], whereas the comparison between *Controls* and *Post-ESL* did not reveal any significant Factor *Group* or *Group* by *Band* interaction [AEC: Factor *Group F*_(1, 60)_ = 3.875, *p* = 0.054, *Group* by *Band* interaction *p* > 0.200; Orthogonalized AEC: Factor *Group F*_(1, 60)_ = 3.351, *p* = 0.064, *Group* by *Band* interaction *p* > 0.200] (Figure 2).

### Clinical and Behavioral Assessment

Twelve patients responded to ESL (54.6%; 6 seizure free) after an average period of treatment of 83.1 ± 19.6 days. None of our patients increased the seizure frequency. The mean seizure reduction was 53.2 ± 42.6% (mean ± standard deviation; median 50%, ranges 0–100%). In the subgroup of patients with available behavioral data, after ESL, sleepiness assessment showed an improvement of ESS (ESS Pre-ESL 6.3 ± 3.1; ESS Post ESL 3.88 ± 2.7; *p* < 0.001) and an unchanged SSS (*p* = 0.08). Depression values were also ameliorated after ESL (BDI, *p* = 0.006) with respect to the Pre.

## Discussion

In the present study we demonstrated that ESL treatment induces cortical EEG activity and connectivity changes in a population of PFE.

The power increase within the epilepsy group was broadband, from delta to gamma. A broadband cortical activity enhancement can depend upon an increased number of neurons firing together or the same group of neurons firing more synchronously (36). The joint increase of activity and connectivity might support a better and stronger engagement of large cortical regions in resting state networks. It is well-known that the epileptic focus modifies the connectivity of the affected cortex with its physiologically-connected regions so that patients with epilepsy show lower global connectivity than controls, with connectivity levels pairing more severe epilepsy conditions (37). We confirmed that patients exhibits lower connectivity levels as compared to controls but–importantly- we also demonstrated that after a 3 months course of eslicarbazepine there was a significant connectivity increase, up to a level very close to that of healthy subjects.

As we observed a selective power increase in theta and gamma band when comparing Post-ESL vs. healthy controls, we could also speculate that ESL exerts a specific action on each frequency band. Although this finding might be a false positive as Pre-ESL theta and gamma activity was slightly higher in epilepsy patients than healthy (Figure 1), we should note that gamma oscillations facilitate synchronization and information transfer, are a positive indicator of brain function and are often associated to clinical improvement (38). Theta (and delta) oscillations can indicate drowsiness (but in our cohort there was no clinical sleepiness increase), augmented cognitive load and network reshaping through long-lasting brain plasticity (36, 39, 40). Finally, the increase of alpha and beta oscillations might improve the cortical resilience to epileptic activity. Alpha oscillations are prominently engaged in visual and attention networks and play an inhibitory role (41–45). Beta activity is largely a residential rhythm of the pyramidal and extra-pyramidal motor network and is considered the player of information gating involved in the maintenance of the status quo of neuronal networks (46, 47).

This study was not designed to compare multiple antiepileptic medications, however we would like to underline that ESL has a similar tolerability profile to recent AED such as levetiracetam (only mild impact on cognition and EEG) and behaves potentially better than carbamazepine and topiramate, which selectively increase delta and theta bands, reduce faster cortical rhythms and more often result in cognitive impairment (11, 13).

Very recently the study of brain connectivity and networks has led to a new conceptualization of focal epilepsy, showing that it is neither involving the entire brain nor a single cortical spot, rather specific brain networks (48). Epileptic activity drives a certain degree of plastic reorganization of brain networks (49). The healthy connections across multiple areas become impaired and patients show increased connectivity of the networks close to the epileptic focus (local networks) and decreased connectivity of long-range networks (37). This re-organization can be clinically relevant, as it can improve the accuracy of the diagnosis (50), help the presurgical planning (51), and represent a marker of the efficacy of epilepsy surgery, as clinical outcome correlates with the degree of restoration of network architecture (52). Rather than be simple proxy of the underlying pathology, network reorganization is therefore a key aspect, that makes focal epilepsy a network disorder. Here we demonstrated a consistent change of cortical connectivity unveiled by three different measures and support the concept of focal epilepsy as a disorder affecting the cortical networks (19).

Future studies should rely on a sample size sufficiently large to allow the characterization of the topographical distribution of the effects in relation to the location of the epileptic focus and to other determinant variables, such as: etiology, disease duration, age (54). This experiment was not directly designed for a clinical application and the results found on group level cannot be applied at single subject level in a clinical setting. Nonetheless, our study demonstrated that simple measures extracted from EEG recording performed in a clinical setting can unveil remarkable effects of medication and, once replicated on larger and better controlled cohorts, might be considered for possible future clinical applications. Such approach could be of translational interest as it might be hypothesized to pursue specific and positive changes of brain activity and connectivity with pharmacological and non-invasive brain stimulation treatments (53, 55, 56).

In conclusion, in this proof-of-principle study we demonstrated that ESL treatment produced an increase in global cortical power and connectivity and encourage the study of quantitative EEG as a potential tool for assessing epilepsy and the effects of antiepileptic medications.

## Author Contributions

GP, OM, PP, MT, VD, and GA: Study design; OM, PP, MT, LR, JL, MB, CD, and GA: Patients recruitment; GP, MT, VD, and GA: Data analysis; GP, GA, OM, VD, and MT: Data interpretation; GP, OM, and GA: Manuscript preparation; All: Manuscript revision.

### Conflict of Interest Statement

The authors declare that the research was conducted in the absence of any commercial or financial relationships that could be construed as a potential conflict of interest.
