# Supplementary material for: Eslicarbazepine Acetate Modulates EEG Activity and Connectivity in Focal Epilepsy
Source: Front Neurol. 2018 Dec 11;9:1054. doi: 10.3389/fneur.2018.01054 (PMC6297144; doi:10.3389/fneur.2018.01054)
Supplement: Supplementary file 1 [file Table_1.docx]

| **Patient N** | **Center** | **Age**  **(yy)** | **Sex** | **Seizure Type** | **Etiology class** | **Etiology** | **lobe** | **Side** | **EEG epileptic**  **activity** | **Epilepsy**  **Duration (years)** | **Therapy at T0 (mg/die)** | **SF/month**  **before ESL** | **SF/month**  **after ESL** |
| --- | --- | --- | --- | --- | --- | --- | --- | --- | --- | --- | --- | --- | --- |
| 1 | I | 45 | M | FIC | S | Hemangiopericytoma | TLE | L | Spikes | 7 | LEV 2000, PHT 100, ZNS 200 | 8 | 1 |
| 2 | I | 53 | M | FIC | S | Head trauma | FLE | L | Spiky theta activity | 26 | PB 200 | 16 | 9 |
| 3 | I | 38 | F | FO | PS |  | TLE | R | Spike and wave | 24 | LTG 350, PB 100 | 2 | 1 |
| 4 | I | 20 | F | FIC | S | Polymicrogyria | FLE | L | Spike and wave | 10 | VPA 300, TPM 400, CBZ 20 | 20 | 4 |
| 5 | I | 53 | M | FO | PS |  | TLE | R | Slow skike and wave | 25 | LTG 100 | 8 | 3 |
| 6 | I | 25 | M | FO | PS |  | PLE | L | Spike and wave | 1 | VPA 1500, CBZ 30 | 2 | 0 |
| 7 | I | 43 | M | FIC | S | Polymicrogyria | TLE | R | Spike and wave | 25 | PB 125 | 6 | 3 |
| 8 | I | 23 | M | FO | PS |  | TLE | R | Spiky theta activity | 3 | VPA 1500, CBZ 10, LEV 2500 | 4 | 0 |
| 9 | I | 41 | M | FIC | S | Cerebral Hemorrhage | TLE | R | Spiky theta activity | 2 | LEV 1500 | 2 | 0 |
| 10 | I | 40 | F | FO | PS |  | TLE | L | Spikes | 38 | VPA 1600, LTG 400, PB 75 | 4 | 4 |
| 11 | I | 37 | M | FO | PS |  | TLE | L | Spikes | 33 | VPA 800, CBZ 20 | 4 | 1 |
| 12 | I | 78 | F | FIC | PS |  | TLE | R | Spiky theta activity | 9 | LEV 2500 | 1 | 1 |
| 13 | II | 42 | F | FIC | S | Low grade Astrocytoma | FLE | R | Spike and wave | 16 | LTG 300, CNZ 2 | 15 | 15 |
| 14 | I | 35 | F | FO | PS |  | TLE | L | Spikes | 15 | TPM 300 | 10 | 10 |
| 15 | I | 69 | F | FO | PS |  | OLE | R | Spike and wave isolated complexes | 35 | PB 150, LEV 500 | 10 | 1 |
| 16 | I | 73 | F | FIC | PS |  | TLE | L | Spiky theta activity | 1 | LEV 1250, CNZ 0.25 | 1 | 0 |
| 17 | I I | 75 | M | FIC | PS |  | TLE | R | Spikes | 6 | PB 100, VPA 500 | 1 | 1 |
| 18 | I | 29 | F | FIC | PS |  | TLE | R | Spiky theta activity | 21 | PB 100, CBZ 600, | 5 | 5 |
| 19 | I | 39 | M | FIC | S | Schwannoma | TLE | R | Spikes | 6 | PER 6, CBZ 600, LTG 300, CLB 10 | 5 | 3 |
| 20 | I | 40 | M | FIC | S | Stroke | TLE | L | Spiky theta activity | 11 | PB 150, LTG 300 | 5 | 1 |
| 21 | I | 70 | M | FIC | S | Epidural hematoma | TLE | L | Spikes | 21 | LTG 200, TPM 200, PB 100 | 6 | 0 |
| 22 | I | 50 | F | FIC | S | Meningioma | TLE | L | Spikes | 2 | LEV 1500 | 1 | 1 |
| **Mean** |  | 46,1 |  |  |  |  |  |  |  | 16,0 |  | 9,9 | 2,4 |
| **Standard Deviation** |  | 16,7 |  |  |  |  |  |  |  | 11,9 |  | 18,4 | 3,0 |

**Table 1. Clinical Feature of the cohort under investigation**

**LEGEND**

**Yy: years**

PS: probably symptomatic (ex cryptogenic); S: structural

FO: focal onset FIC: focal onset with impaired consciousness

M: male; F: Female

TLE: temporal lobe epilepsy; FLE: frontal lobe epilepsy; PLE: parietal lobe epilepsy; OLE: occipital lobe epilepsy;

L: left; R: right

SF: seizure frequency

I: Università La Sapienza, Policlinico Umberto I

II: Campus Bio-Medico University of Rome.

LEV=levetiracetam, PHT=Phenytoin, ZNS=Zonisamide, PB=Phenobarbital, LTG=Lamotrigine, VPA=Valproic Acid, TPM=Topiramate, CLB=Clobazam, CNZ=clonazepam, CBZ=Carbamazepine, PER=Perampanel
